# Evaluation of *Meloidogyne incognita* and *Rotylenchulus reniformis* nematode-resistant cotton cultivars with supplemental Corteva Agriscience nematicides

**DOI:** 10.2478/jofnem-2023-0001

**Published:** 2023-02-17

**Authors:** A. Kate Turner, Scott H. Graham, Neha Potnis, Steve M. Brown, Pat Donald, Kathy S. Lawrence

**Affiliations:** 559 Devall Dr. CASIC Building, Auburn Univ, AL 36849 Alabama United States; ALFA Building, Auburn Univ, AL 36849 Alabama United States; 209 Life Science Building, Auburn Univ, AL 36849 Alabama United States; 249 Funchess Hall Auburn Univ, AL 36849 Alabama United States

**Keywords:** cotton, integrated nematode management, *Meloidogyne*, root-knot nematode, *Rotylenchulus*, reniform nematode

## Abstract

*Meloidogyne incognita*- and *Rotylenchulus reniformis*-resistant new cotton cultivars have recently become available, giving growers a new option in nematode management. The objectives of this study were: (i) to determine the yield potential of the new cultivars PHY 360 W3FE (*M. incognita*-resistant) and PHY 332 W3FE (*R. reniformis*-resistant) in nematode-infested fields and (ii) to evaluate the effects of combining the nematicides Reklemel (fluazaindolizine), Vydate C-LV (oxamyl), and the seed treatment BIO^ST^ Nematicide 100 (heat killed *Burkholderia rinojenses* and its non-living spent fermentation media) with resistant cotton cultivars on nematode population levels and lint yield. Field experiments in 2020 and 2021 indicated *M. incognita* population levels were 73% lower on PHY 360 W3FE (R) and 80% lower for *R. reniformis* on the PHY 332 W3FE (R) at 40 days after planting. Nematode eggs per gram of root were further reduced an average of 86% after the addition of Reklemel and Vydate C-LV when averaging both cultivars over the two years. Tests with BIO^ST^ Nematicide 100 + Reklemel + Vydate C-LV (0.56 + 2.5 L/ha) in both *M. incognita* and *R. reniformis* fields produced higher lint yields. Overall, planting PHY 360 W3FE (R) and PHY 332 W3FE (R) improved yields an average of 364 kg/ha while limiting nematode population increases. The addition of the nematicides further increased yields 152 kg/ha of the nematode-resistant cultivars.

Upland cotton, *Gossypium hirsutum*, (L.) is one of the most important cash crops in US production and is the leading natural fiber cash crop exported in the US (Ma et al., 2018; USDA, 2020). Upland cotton accounts for 90% of the world’s cotton production, and 97% of production in the United States (Cotton Incorporated, 2020; USDA, 2020). *M. incognita* (Kofoid and White) Chitwood and *Rotylenchulus reniformis* (Linford and Oliveira) are major, yield-limiting pests of upland cotton, (Lawrence et al., 2021). *M. incognita* is one of the most important plant parasitic nematodes globally because of its ability to damage agricultural crops and cultivated plants (Wram and Zasada, 2019). *M. incognita* accounts for an estimated annual yield loss of over $100 billion worldwide, and $283 million for cotton in the United States (Forghani and Hajihassani, 2020; Lawrence et al., 2022). In Alabama, *M. incognita* is estimated to reduce cotton yield 4.5%, which is equivalent to 34,000 bales of cotton (Lawrence et al., 2022). Management of *M. incognita* in cotton production relies heavily on nematicides due to its wide host range and the limited number of resistant cultivars (Starr et al., 2007; Davis and Kemerait, 2022). Breeding for *Meloidogyne* spp. nematode resistance in cotton began in the early 1900s, but it was not until 1953 when the first cotton cultivar, Auburn 56, was developed with moderate resistance to *M. incognita* (Smith, 1964; Zhang et al., 2006). The first highly cotton germplasm line highly resistant to *M. incognita* was Auburn 623 RNR, released in March 1970 by Auburn University Agricultural Experiment Station and the Agricultural Research Service (ARS) of the USDA (Shepherd and Huck, 1974). In cotton, more than one dominant resistant gene provide a high level of resistance, while recessive genes provide a moderate level of resistance to *M. incognita*. It is believed that these resistance genes are associated with chromosomes 11 and 14 (Gutierrez et al., 2010; Gaudin and Wubben, 2021). Chromosome 11 is indicated to suppress the formation of root galls, while chromosome 14 is indicated to suppress egg production (He et al., 2014). Breeding efforts have been continuous since the 1970s to produce highly *M. incognita*-resistant cotton cultivars with better agronomic characteristics than Auburn 623 RNR (McPherson et al., 2004). Currently, there are at least 10 cotton cultivars with one or two genes for resistance to *M. incognita* available through Corteva Agriscience, Bayer, and BASF (Kichler, 2021). Cotton cultivars that have some form of resistance to *M. incognita* are listed in Table 1 (Weaver, 2015; Corteva Agriscience, 2018; Wheeler et al., 2020).

**Table 1:** Cotton (*Gossypium hirsutum*) cultivars grouped into *M. incognita* (*Mi*) or *R. reniformis* (*Rr*) resistance categories by company.

Cultivar	*Mi* resistance	*Rr* resistance	Company
Fibermax 1621 GL	PRa		BASF; Ludwigshafen, Germany
Fibermax 1911 GLT	PR		BASF; Ludwigshafen, Germany
Fibermax 2011 GT	PR		BASF; Ludwigshafen, Germany
Fibermax 1730 GLTP	PR		BASF; Ludwigshafen, Germany
Stoneville 4946 GLB2	PR		BASF; Ludwigshafen, Germany
Stoneville 5600 B2XF	PR		BASF; Ludwigshafen, Germany
Deltapine 2141 NR B3XF	PR	PR	Bayer; Leverkusen, Germany
Deltapine 2143 NR B3XF	PR	PR	Bayer; Leverkusen, Germany
Deltapine 1454NR B2RF	NRb		Bayer; Leverkusen, Germany
Deltapine 1558NR B2RF	NR		Bayer; Leverkusen, Germany
Deltapine 1747NR B2RF	NR		Bayer; Leverkusen, Germany
Deltapine 1823NR B2XF	NR		Bayer; Leverkusen, Germany
PhytoGen 250 W3FE	PR		Corteva AgriScience; Indianapolis, IN
PhytoGen 332 W3FE	Rc	R	Corteva AgriScience; Indianapolis, IN
PhytoGen 350 W3FE	PR		Corteva AgriScience; Indianapolis, IN
PhytoGen 390 W3FE	R		Corteva AgriScience; Indianapolis, IN
PhytoGen 400 W3FE	PR		Corteva AgriScience; Indianapolis, IN
PhytoGen 430 W3FE	PR		Corteva AgriScience; Indianapolis, IN
PhytoGen 440 W3FE	PR		Corteva AgriScience; Indianapolis, IN
PhytoGen 443 W3FE	R	R	Corteva AgriScience; Indianapolis, IN
PhytoGen 545 W3FE	R		Corteva AgriScience; Indianapolis, IN
PhytoGen 320 W3FE	PR		Corteva AgriScience; Indianapolis, IN
PhytoGen 360 W3FE	R		Corteva AgriScience; Indianapolis, IN
PhytoGen 417 WRF	HRd		Corteva AgriScience; Indianapolis, IN
PhytoGen 427 W3FE	R		Corteva AgriScience; Indianapolis, IN
PhytoGen 480 W3FE	HR		Corteva AgriScience; Indianapolis, IN
PhytoGen 530 W3FE	R		Corteva AgriScience; Indianapolis, IN
PhytoGen 580 W3FE	HR		Corteva AgriScience; Indianapolis, IN

^a^ PR are cultivars reported with partial resistant to *M. incognita* or *R. reniformis*. ^b^ NR are cultivars reported to be resistant to *M. incognita* or *R. reniformis*. ^c^ R are cultivars reported to have some form of resistance, but not fully evaluated yet. ^d^ HR are cultivars reported to have high resistance with two homologous resistant genes.

*R. reniformis* is one of the most economically important plant-parasitic nematodes of upland cotton (Gordon et al., 2022). *R. reniformis* reportedly caused an estimated annual loss of approximately $33 million dollars in the United States in 2020 (Wilson et al., 2020). In Alabama, *R. reniformis* causes an estimated 3% of cotton yield loss in 2021, which was approximately 22,700 bales of cotton (Lawrence et al., 2022). Previously, management of *R. reniformis* consisted of integrated practices of crop rotation, cover cropping, and nematicides (Davis et al., 2003; Wilson et al., 2020). The screening for *R. reniformis*-resistant cotton cultivars began in the early 1960s with greenhouse tests done by Birchfield and Brister (Khanal et al., 2018). All upland cotton (*G. hirsutum*) cultivars that have been evaluated are susceptible to *R. reniformis* nematode; however, *R. reniformis* resistance has been detected in other species of cotton (Bhandari et al., 2015). *R. reniformis* resistance has been identified in at least 10 of 50 cotton species, including G. *anomalum* (Waw. and Peyr), G. *arboretum* L., G. *aridum* (Rose & Standley) Skovsted, G. *herbaceum* L., G. *raimondii* Ulbrich, G. *somalense* (Gürke) Hutch, G. *stocksii*, G. *thurberi* Tod, G. *longicalyx*, (Hutch and Lee) and G. *barbadense L*. (Yik and Birchfield, 1984; Li et al., 2018). Among these, only G. *longicalyx* displayed immunity to *R. reniformis* (Li et al., 2018). In other species of cotton, six *R. reniformis* resistance genes have been identified, ranging from partial dominant, dominant, and recessive in action (Khanal et al., 2018). *Ren^lon^* and *Ren^ari^* genes are dominant and identified in *G. longicalyx* on chromosome 11, *G. aridum* on chromosome 21, and *Ren^barb^*1, *Ren^barb^*2, and *Ren^barb3^* are partially resistant and identified in *G. barbadense*, located on chromosomes 21 and 18, respectively (Khanal et al., 2018). Multiple upland cotton cultivars such as Stoneville 4793 (BASF, Ludwigshafen, Germany), Suregrow 521 R, Suregrow 215 BR, Paymaster 1218 BR, and Deltapine 449 BR (Bayer, Leverkusen, Germany) showed potential tolerance to *R. reniformis* infection, but cultivars were inconsistent in their ability to tolerate *R. reniformis* (Blessitt et al., 2012). Bayer CropScience released DP 2141 NR B3XF and DP 2143 NR B3XF, reporting partial resistance to *R. reniformis* while PhytoGen seed company is releasing their first *R. reniformis*-resistant cultivars PHY 332 W3FE and PHY 433 W3FE (Boyd, 2020). The two PhytoGen *R. reniformis*-resistant cultivars contain three genes for resistance, which include two genes for resistance to *M. incognita* and an additional gene that adds the *R. reniformis* resistance. The cotton cultivars with *R. reniformis* resistance are listed in Table 1 (Weaver, 2015; Corteva Agriscience, 2020; Wheeler et al., 2020). High-yielding cotton cultivars often are planted on large percentages of the acres without rotations. Concern in the cotton community is that repeated planting of a nematode-resistant cultivar may eventually lead to natural selection of resistance in the nematode population, negating nematode resistance in the cotton cultivar (Young, 1992; Davis and Kemerait, 2009).

New nematicides and nematicide combinations provide additional management tools. The seed treatment BIO^ST^ Nematicide 100 (Albaugh, Ankeny, IA) is labeled to protect cotton against nematodes. BIO^ST^ Nematicide 100 is a biological nematicide derived from heat-killed *Burkholderia rinojenses* and its non-living spent fermentation media with multiple modes of action via enzymes and toxins (Albaugh, 2016). Reklemel, fluazaindolizine, is a new nematicide against *M. incognita* and *R. reniformis* (Lahm et al., 2017; Talavera et al., 2021), and it has been shown to significantly reduce motility and activity of *M. incognita* and *R. reniformis* (Lahm et al. 2017; Groover and Lawrence, 2021). Reklemel is currently labeled for use in vegetable crops to reduce nematode damage and enhance yields (Desaeger, 2022). Vydate C-LV is an older foliar-applied nematicide/insecticide that provides management of *M. incognita* and *R. reniformis* in cotton (David and Kemerite, 2022; Lawrence, 2022) and has been shown to be effective against *M. incognita* and *R. reniformis* through suppression of nematode population and feeding (Lawrence and McLean, 2000; Mueller et al., 2012). Neither Reklemel nor Vydate C-LV have been applied in-furrow at planting on cotton for the management of *M. incognita* and *R. reniformis* in cotton. The combination of selected nematicides and nematode-resistant cotton cultivars could potentially extend the seasons a resistant cultivar could be produced without requiring a crop rotation to manage the nematode pests (Davis and Kemerait, 2009).

We hypothesized that combining the use of nematode resistant cotton cultivars with additional seed treatment and in-furrow nematicides would provide for an integrated nematode management system for *M. incognita* and *R. reniformis* in cotton production. The purpose of this study was to compare susceptible (S) and resistant (R) cotton cultivars PHY 340 W3FE (S), and PHY 360 W3FE [*M. incognita* (R)] and PHY 332 W3FE [*R. reniformis* (R)], and the effects of additional new nematicide combinations on *M. incognita* and *R. reniformis* population development and yield in upland cotton. The objectives of this study were i) to determine the cultivar effect on the nematode populations development in controlled greenhouse conditions; ii) determine the yield potential of the *M. incognita* resistant variety PHY 360 W3FE and the *R. reniformis* resistant variety PHY 332 W3FE in nematode infested fields; and iii) to evaluate the effects of combining the new nematicide Reklemel (fluazaindolizine), with Vydate C-LV (oxamyl), and the seed treatment BIO^ST^ Nematicide 100 (heat-killed *Burkholderia rinojenses* and its non-living spent fermentation media) with the cotton genetic resistances on nematode population levels and subsequent cotton lint yield.

## Materials and Methods

Greenhouse experiments were established to evaluate the population density of *M. incognita* and *R. reniformis* on the cotton cultivars PHY 340 W3FE (S), PHY 360 W3FE *M. incognita* (R), and PHY 332 W3FE *R. reniformis* (R). Cultivar names will be abbreviated to PHY 340 (S), PHY 360 (R), and PHY 332 (R) hereafter. Duplicate field experiments were established to determine nematode population development on the PHY 340 (S) compared to the PHY 360 *M. incognita* (R) in a *M. incognita*-infested cotton field. PHY 340(S) was compared to PHY 332 *R. reniformis* (R) in a separate *R. reniformis*-infested cotton field. Nematicide applications included the seed treatment BIO^ST^ Nematicide 100 (0.026 mg ai/seed) or no-seed treatment nematicide. Additional nematicides Reklemel + Vydate C-LV combination applied as an in-furrow spray at planting at three rates of (0.28 + 1.24 L/ha), (0.56 + 2.5 L/ha), or (1.13 + 5.0 L/ha) to evaluate the interaction of the genetics of the nematode-resistant cultivars PHY 360 or PHY 332 with nematicides. The Reklemel + Vydate C-LV in-furrow spray rates were reduced in the second year of testing to (0.21 + 0.88 L/ha), (0.28 + 1.24 L/ha), and (0.56 + 2.5 L/ha). A complete list of the field treatments is listed in Table 2.

**Table 2:** Cotton cultivar PHY 340 W3FE [susceptible(S)], PHY 360 W3FE [*M. incognita* resistant(R)] and PHY 332 W3FE [*R. reniformis* resistant (R)] and nematicide rates and combinations used in field experiments conducted at the Plant Breeding Unit (PBU), Shorter, AL and Tennessee Valley Research and Extension Center (TVREC), Belle Mina, AL in 2020 and 2021.

Number	Cotton cultivar	Nematicides	2020 rates	2021 rates	Application
1	PHY 340 W3FE (S)	No nematicide			
2	PHY 340 W3FE (S)	Reklemel^a^ + Vydate C-LV^b^	0.28 + 1.24 L/ha	0.21 + 0.88 L/ ha	In-furrow spray
3	PHY 340 W3FE (S)	Reklemel + Vydate C-LV	0.56 + 2.5 L/ha	0.28 + 1.24 L/ ha	In-furrow spray
4	PHY 340 W3FE (S)	Reklemel + Vydate C-LV	1.13 + 5.0 L/ha	0.56 + 2.50 L/ ha	In-furrow spray
5	PHY 340 W3FE (S)	BIO^ST^ Nematicide 100^c^	0.026 mg ai/seed	0.026 mg ai/ seed	Seed treatment
6	PHY 340 W3FE (S)	BIO^ST^ Nematicide 100 + Reklemel + Vydate C-LV	0.026 mg ai/seed + 0.28 + 1.24 L/ ha	0.026 mg ai/ seed + 0.21 + 0.884 L/ha	Seed treatment + In-furrow spray
7	PHY 340 W3FE (S)	BIO^ST^ Nematicide 100 + Reklemel + Vydate C-LV	0.026 mg ai/seed + 0.56 + 2.50 L/ ha	0.026 mg ai/ seed + 0.28 + 1.24 L/ha	Seed treatment + In-furrow spray
8	PHY 340 W3FE (S)	BIO^ST^ Nematicide 100 + Reklemel + Vydate C-LV	0.026 mg ai/seed + 1.13 + 5.0 L/ha	0.026 mg ai/ seed + 1.13 + 5.0 L/ha	Seed treatment + In-furrow spray
9	PHY 360 W3FE (R) or PHY 332 W3FE (R)^c^	No nematicide			
10	PHY 360 W3FE (R) or PHY 332 W3FE (R)	Reklemel + Vydate C-LV	0.28 + 1.24 L/ha	0.21 + 0.88 L/ ha	In-furrow spray
11	PHY 360 W3FE (R) or PHY 332 W3FE (R)	Reklemel + Vydate C-LV	0.56 + 2.5 L/ha	0.28 + 1.24 L/ ha	In-furrow spray
12	PHY 360 W3FE (R) or PHY 332 W3FE (R)	Reklemel + Vydate C-LV	1.13 + 5.0 L/ha	0.56 + 2.50 L/ ha	In-furrow spray
13	PHY 360 W3FE (R) or PHY 332 W3FE (R)	BIO^ST^ Nematicide 100^e^	0.026 mg ai/seed	0.026 mg ai/ seed	Seed treatment
14	PHY 360 W3FE (R) or PHY 332 W3FE (R)	BIO^ST^ Nematicide 100 + Reklemel + Vydate C-LV	0.026 mg ai/seed + 0.28 + 1.24 L/ ha	0.026 mg ai/ seed + 0.21 + 0.88 L/ha	Seed treatment + In-furrow spray
15	PHY 360 W3FE (R) or PHY 332 W3FE (R)	BIO^ST^ Nematicide 100 + Reklemel + Vydate C-LV	0.026 mg ai/seed + 0.56 + 2.50 L/ ha	0.026 mg ai/ seed + 0.28 + 1.24 L/ha	Seed treatment + In-furrow spray
16	PHY 360 W3FE (R) or PHY 332 W3FE (R)	BIO^ST^ Nematicide 100 + Reklemel + Vydate C-LV	0.026 mg ai/seed + 1.13 + 5.0 L/ha	0.026 mg ai/ seed + 0.56 + 5.0 L/ha	Seed treatment + In-furrow spray

^a^ Reklemel active nematicide is 500g/L Fluazaindolizine. ^b^ Vydate C-LV active nematicide is 452 g/L Oxamyl. ^c^ Heat-killed *Burkholderia rinojenses* and its non-living spent fermentation media.

*Nematode Inoculum and Extraction: M. incognita* was maintained on corn *Zea mays* L. “Pioneer 1197 YHR” (Corteva Agriscience, Wilmington, DE) in 2020 and “Pioneer 1506 YHR” in 2021 and *R. reniformis* was maintained on cotton “Deltapine 1646 B2XF” (Bayer AG, Leverkusen, Germany) in 2020 and “PhytoGen 340 W3FE” (Corteva Agriscience, Wilmington, DE) in 2021 in 500 cm^3^ polystyrene cups (Dart Container Corporation, Mason, Michigan) at the Plant Science Research Center (PSRC) in Auburn, AL for inoculum. Eggs of *M. incognita* and *R. reniformis* were extracted by the modified root extraction method of Hussey and Barker (1973). The tops of the infected plant were removed, and the roots were gently washed in water to remove excess soil. Roots were then placed in a 0.625% NaOCl solution and shaken for 4 min at one g-force on a Barnstead Lab Line Max Q 5000E Class shaker (Thermo Fisher Scientific, Waltham, MA). The eggs were collected on a 25-μm pore sieve, rinsed with water, and washed into 50 mL centrifuge tubes. The contents were mixed with a 1.14-specific gravity sucrose solution and centrifuged at 1400 g-forces for 1 min (Jenkins, 1964). After centrifugation, the eggs in the supernatant of the sucrose solution were collected on a 25-μm pore sieve and rinsed with water to remove the sucrose solution from the eggs. *M. incognita* and *R. reniformis* egg were enumerated using a Nikon TSX 100 inverted microscope (Brighton, MI at 40x magnification and adjusted to 5,000 eggs/mL.

*Greenhouse Experiments*: Separate *M. incognita* and *R. reniformis* greenhouse tests were conducted at PSRC to determine *M. incognita* population density increases on PHY 340 (S) and PHY 360 (R) and *R. reniformis* population density increases on PHY 340 (S) and PHY 332 (R) (Corteva Agriscience, Wilmington, DE) cotton, respectively. All seeds were pretreated with the insecticide/fungicide seed treatments metalaxyl 4.0 ST, fludioxonil 4L ST, myclobutanil 240 ST, and imidacloprid. A Kalmia loamy sand texture soil (80% sand, 10% silt, and 10% clay; 1.2% organic matter, pH 6.9) from the Plant Breeding Unit (PBU) near Tallassee, AL was pasteurized at 88°C for 12 hr then allowed to cool for 24 hr, and the process was repeated. The pasteurized soil was mixed with sand at a rate of 60:40 soil to sand. Fertilizer and lime were added to the soil at rates recommended by the soil analysis. Seeds were planted in 500-cm^3^ polystyrene cups. Four cotton seeds and 5,000 nematode eggs were added per pot at a depth of 2.5 cm at the initiation of the experiments. All plots were arranged in a randomized complete block design (RCBD) with five replications, and each test was repeated each year for a total of eight tests over the two years. Plants were watered as needed to maintain soil moisture, and lighting was supplied via 1,000-watt halide bulbs producing 110,000 lumens for a 14-hr day length. Temperature in the greenhouse ranged from 25°C to 29°C.

Data were collected at 30 d after planting (DAP) for all greenhouse experiments. Plant parameters included plant stand, (PS), plant height (PH), shoot fresh weight (SFW), root fresh weight (RFW), and biomass (SFW + RFW). Nematode parameters of *M. incognita* and *R. reniformis* population density included the total number of eggs extracted from the roots and reported on a per gram of root basis. Greenhouse tests were conducted in 2020 with the PhytoGen seeds lots used in the field experiments that year. In 2021, new PhytoGen seeds were acquired for the second year of field tests; thus the greenhouse tests were repeated with the new seed lots.

*Field Experiments*: Field experiments were conducted at two locations: PBU and Tennessee Valley Research Extension Center (TVREC) near Belle Mina, AL. PBU is naturally infested with *M. incognita* race 3 and initial at-plant levels were 222 vermiform life stages per 100 cm^3^ of soil in 2020 and 508 vermiform life stages per 100 cm^3^ of soil in 2021. PBU has a soil type of Kalmia loamy sand, previously described. TVREC was artificially infested in 2007 with *R. reniformis*. For TVREC, the population density at planting in 2020 was 5,000 vermiform life stages per 100 cm^3^ of soil and 1,158 vermiform life stages per 100 cm^3^ of soil in 2021. TVREC soil type is a Decatur silt loam, which consists of 23% sand, 49% silt, and 28% clay; 1% organic matter, pH 6.0. Field test plots at both locations consisted of two rows, 7.6 m long, with a 1-m row spacing and a 4.6-m alley between replications. All experiments were placed in a factorial arrangement of an RCBD with 10 replications, and each plot was planted with 13 seeds per meter of row at a depth of 2 cm using a John Deere MaxEmerge (John Deere, Moline, IL) planter with Almaco cone planters (Almaco, Nevada, IA).

Nematicides included a seed treatment and in-furrow spray applications. BIO^ST^ Nematicide 100 seed treatment was applied by Corteva at 0.026 mg ai/seed. At planting, an in-furrow spray application of Reklemel and Vydate C-LV (Table 2) were applied in the furrow directly behind the seed at 30 PSI using 8,002 flat fan nozzles at PBU and a 30-PSI orifice at TVREC. In-furrow nematicide applications were made at rates of Reklemel 0.28 L/ha (140 g/ ha), 0.56 L/ha (280 g/ha), and 1.13 L/ha (560 g/ ha), combined with Vydate C-LV at 1.24 L/ha (560 g/ha), 2.5 L/ha (1120 g/ha), and 5.0 L/ha (2240 g/ ha) in 2020. In 2021, in-furrow spray applications were reduced and applied at 0.21 L/ha (110 g/ha), 0.28 L/ha (140 g/ha), and 0.56 L/ha (280 g/ha) for Reklemel, and 0.88 L/ha (392 g/ha), 1.24 L/ha (560 g/ha), and 2.5 L/ha (1120 g/ha) for Vydate C-LV. Field plots were irrigated with a center pivot sprinkler system at PBU and a lateral irrigation system at TVREC as needed throughout the growing season. Planting and harvest dates in 2020 were May 7 and October 7 at PBU, and May 5 and October 21 at TVREC. PBU and TVREC sites in 2021 were planted April 27 and May 7 and harvested October 20 and November 8, respectively.

*Data collection*: Plant parameters included plant stand (PS) at 30 DAP. PH, SFW, RFW, biomass, and nematode eggs per gram of root were collected at 40 DAP. In the *M. incognita* experiments, root-gall ratings on a scale of 0-10 (0 having no galls on roots and 10 all roots severely galled) (Bridge and Page, 1980) were recorded at 40 DAP and immediately after harvest. Plant parameters and nematode populations were obtained from four random representative cotton plants per plot. Plants were transported from the field to PSRC, and nematode population density was determined as described in the nematode inoculum section for *M. incognita* and *R. reniformi*s. Plots were machine-harvested for yield with an Almaco SPC40 plot combine (Nevada, IA).

*Statistical analysis*: Data collected from the greenhouse experiments were analyzed in SAS 9.4 (SAS Institute, Cary, NC) using the PROC GLIMMIX. Tukey Kramer LS-means were compared using ANOVA at a significance level of *P* ≤ 0.05. Dependent variables were PS, PH, SFW, RFW, biomass, gall ratings, *M. incognita* and *R. reniformis* total egg numbers (eggs/root), *M. incognita* and *R. reniformis* eggs per gram of root (eggs/g of root). Nematode eggs/root and eggs/g of root were log-transformed to fulfill the normal assumption and back-transformed for presentation. There were no significant interactions in the greenhouse tests between 2020 and 2021; thus the data from both years were pooled into a single data set.

Data collected from field experiments were analyzed in SAS 9.4 (SAS Institute, Cary, NC) using the PROC GLIMMIX. A two-way factorial ANOVA analysis was conducted with cultivar, nematicides, and cultivar x nematicides for each year of the experiments. Dependent variables were PS, PH, SFW, RFW, biomass, gall ratings, *M. incognita* and *R. reniformis* total egg numbers (eggs/root), *M. incognita* and *R. reniformis* eggs per gram of root (eggs/g of root), and lint yield. Means were separated using Tukey Kramer LS-means test at the *P* ≤ 0.05 level. Student panels were produced to determine the normality of the residuals. Nematode eggs/g of root data were log-transformed to satisfy the ANOVA assumptions of normally distributed residuals and back-transformed for presentation on the tables. Repeated tests were conducted each year and combined for 10 repetitions for each test each year, or 160 experimental units for each nematode, *M. incognita* and *R. reniform*, each year. Pearson’s correlation coefficient (PROC CORR) was used to determine relationships between nematode eggs/g of root and lint yield.

## Results

*Greenhouse nematode tests*: In the greenhouse setting with *M. incognita*, both PHY 360 (R) and PHY 340 (S) cultivars had similar PH, SFW, and biomass (Table 3). However, RFW was greater (*P* < 0.0024) on the PHY 340 (S) cotton. *M. incognita* total egg numbers and eggs per gram of root density were significantly lower (*P* < 0.0009) on PHY 360 (R) cultivar compared to PHY 340 (S). PHY 360 (R) supported 90% fewer *M. incognita* total eggs as compared to PHY 340 (S). When placed on the per gram of root basis, the pattern was similar with 88% fewer *M. incognita* eggs per gram of root supported on PHY 360 (R) compared to PHY 340 (S).

**Table 3:** Greenhouse trial LS means for PHY 360 W3FE, *M. incognita* resistant (R), PHY 332 W3FE, *R. reniformis* resistant (R), each paired with PHY 340 W3FE, susceptible (S) cotton variety effects on plant height, shoot fresh weight, root fresh weight, biomass, total nematode eggs, and nematodes eggs per gram of root at 30 DAP at the Plant Science Research Center, Auburn, AL in 2020 and 2021.

Cotton cultivar	Plant height (cm)	Shoot fresh weight (g)	Root fresh weight (g)	Biomass (g)	Total *M. incognita* eggs	*M. incognita* eggs/g of root^a^
PHY 360 W3FE	12.63 A	6.55 a	4.31 b	10.85 a	194 b	42 B
PHY 340 W3FE^b^	11.95 a^c^	6.21 a	6.68 a	12.89 a	1922 a	353 A
*P*-values	0.4848	0.6348	0.0024	0.0749	0.0001	0.0009
**Cotton cultivar**	**Plant height (cm)**	**Shoot fresh weight (g)**	**Root fresh weight (g)**	**Biomass (g)**	**Total *R. reniformis* eggs**	***R. reniformis*** **eggs/g of root**
PHY 332 W3FE	11.02 A	5.5 a	4.57 a	10.06 a	447 b	146 A
PHY 340 W3FE	11.97 A	6.24 a	6.55 a	12.79 a	1935 a	358 A
*P*-values	0.3803	0.3223	0.0797	0.0871	0.0003	0.0810

^a^Data for *M. incognita and R. reniformis* eggs/gram of root were collected from four root systems. ^b^All seeds were treated with Metalaxyl 4.0 ST, Fludioxonil 4L ST, Myclobutanil 240 ST, Resonate 600. ^c^Values present are LS-means separated using the Tukey-Kramer method at *P* ≤ 0.05. Values in the same column followed by the same letter do not differ significantly. *P*-values listed are type III fixed effects.

In the greenhouse setting with *R. reniformis*, both PHY 332 (R) and PHY 340 (S) cultivars had similar PH, SFW, RFW, and biomass (Table 3). *R. reniformis* total eggs and eggs per gram of root were significantly lower (*P* < 0.003 and *P* < 0.0810) on PHY 332 (R) compared to PHY 340 (S). PHY 322 (R) supported 77% fewer *R. reniformis* total eggs as compared to PHY 340 (S).

**Figure 1:**
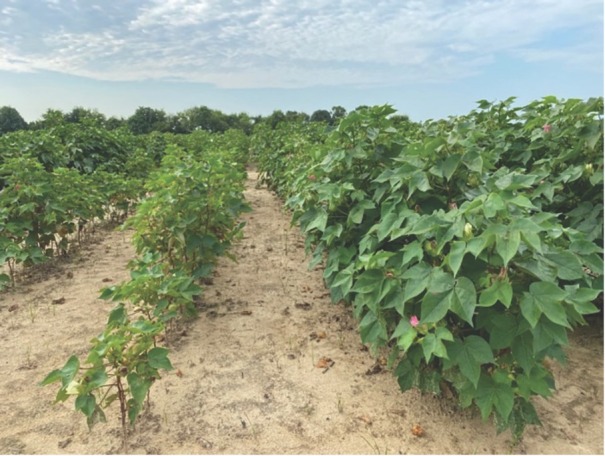
*M. incognita* PHY 340 W3FE susceptible variety on the left and the resistant PHY 360 W3FE on the right at 106 DAP.

*M. incognita Field 2020*: The factorial analysis in PROC GLIMMIX indicated there were no significant interactions between the cotton cultivars and nematicides for the plant parameters (Table 4). Plant stand was greater for the PHY 340 (S) with 9.4 plants per meter of row surviving compared to 5.8 plants per meter of row for PHY 360 (R) (Table 4). PH and RFW were similar between both cotton cultivars and all nematicide applications. Biomass was significantly greater, with PHY 340 (S) weighing 0.5 g more than PHY 360 (R) (Table 4, Fig. 1). There was a significant positive correlation between biomass and lint yield (r = 0.23025; *P* < 0.0034), indicating an increase in plant growth overall leads to an increase in lint yield. PHY 360 (R) supported a significantly higher lint yield than PHY 340 (S). Lint yield was increased by 92 kg/ ha or 6% when growing PHY 360 (R) compared to PHY 340 (S) in an *M. incognita* field.

**Table 4:** Field trial LS means for PHY 360 W3FE [*M. incognita* resistant (R)] and PHY 340 W3FE [*M. incognita* susceptible (S)] cotton variety and nematicide combination effects on plant stand, plant height, root fresh weight, plant biomass, and lint cotton yield at the Plant Breeding Unit, Shorter, AL in 2020.

Source of variation (F-value)	Plant stand (13 m row)	Plant height (cm)	Root fresh weight (g)	Biomass (g)	Lint cotton yield (kg/ha)
Cultivar^a^	0.0001^****b^	0.5451	0.1338	0.0206^*^	0.0402^**^
Nematicide^c^	0.0001^****^	0.1340	0.3412	0.1491	0.001^***^
Cultivar x Nematicide	0.3891	0.9234	0.9204	0.9481	0.3753
**Cultivar LS-means**					
PHY 360 W3FE (R)	44.58^d^ b	13.91 a	0.49 a	4.57 b	1621 a
PHY 340 W3FE (S)	71.58 a	13.69 a	0.52 a	5.06 a	1529 b
**Nematicide LS-means**					
No nematicide	63.25 a	14.13 a	0.50 a	4.74 a	1527 ab
Reklemel (0.28 L/ha) + Vydate C-LV (1.24 L/ha)	60.65 a	14.79 a	0.52 a	5.17 a	1489 ab
Reklemel (0.56 L/ha) + Vydate C-LV (2.5 L/ha)	45.65 b	13.43 a	0.49 a	4.22 a	1363 b
Reklemel (1.13 L/ha) + Vydate C-LV (5.0 L/ha)	48.00 b	13.20 a	0.54 a	4.77 a	1458 ab
BIO^ST^ Nematicide 100^e^ (0.026 mg ai/seed)	61.35 a	12.74 a	0.47 a	4.41 a	1657 a
BIO^ST^ Nematicide 100 (0.026 mg ai/seed) + Reklemel (0.28 L/ha) + Vydate C-LV (1.24 L/ha)	63.45 a	14.15 a	0.51 a	5.33 a	1716 a
BIO^ST^ Nematicide 100(0.026 mg ai/seed) + Reklemel (0.56 L/ha) + Vydate C-LV (2.5 L/ha)	62.70 a	13.74 a	0.50 a	4.81 a	1720 a
BIO^ST^ Nematicide 100 (0.026 mg ai/seed) + Reklemel (1.13 L/ha) + Vydate C-LV (5.0 L/ha)	59.55 a	14.25 a	0.53 a	5.08 a	1671 a

^a^All seeds were treated with Metalaxyl 4.0 ST, Fludioxonil 4L ST, Myclobutanil 240 ST, Resonate 600. ^b^*P*-values for Type III fixed effects with significance at the 0.1, 0.05, 0.01, and 0.001 level are indicated by *, **, ***, and ****, respectively. ^c^Reklemel and Vydate C-LV were applied at planting as an in-furrow spray and BIO^ST^ Nematicide 100 was a seed treatment previously added to the seeds. ^d^Values present are LS-means separated using the Tukey-Kramer method at *P* ≤ 0.05. Values in the same column followed by the same letter do not differ significantly.

Nematicide combinations of Reklemel + Vydate C-LV at the rates (0.56 + 2.5 L/ha) and (1.13 + 5.0 L/ ha), respectively, had a significantly lower plant stand compared to all other nematicide combinations. The nematicide combinations did not significantly affect PH, RFW, or biomass. The BIO^ST^ Nematicide 100 seed treatment alone or combined with all rates of Reklemel (0.28, or 0.56, or 1.13 L/ha) + Vydate C-LV (1.24, or 2.5, or 5.0 L/ha) supported the highest lint yields compared to the Reklemel + Vydate C-LV (0.56 + 2.5 L/ha) alone. Lint yield was increased by an average of 164 kg/ha or 9% with the addition of the BIO^ST^ plus Reklemel and Vydate C-LV nematicide across all rates compared to the no-nematicide control.

**Figure 2:**
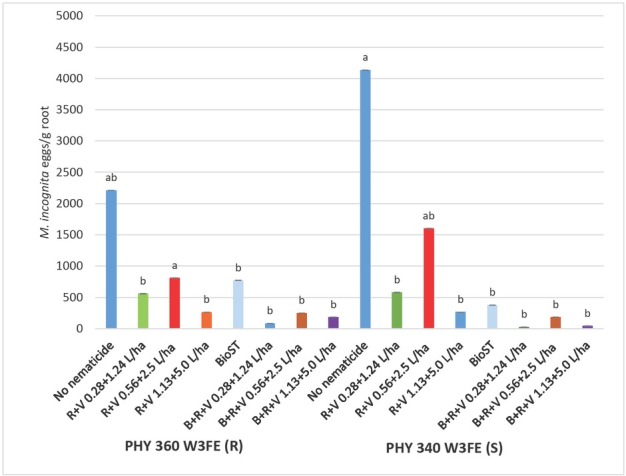
Field trial *M. incognita* eggs per gram of root collected from four root systems LS means for PHY 360 W3FE (R) and PHY 340 W3FE (S) cotton and nematicide combination at 40 DAP in 2020. *P-*value for Type III fixed effects for the Variety x Nematicide interaction was 0.0368. Nematicide treatments included no-nematicide control, Reklemel (0.28 L/ha) + Vydate C-LV (1.24 L/ha), C-LV (0.28 +1.24 L/ha), Reklemel (0.56 L/ha) + Vydate C-LV (2.5 L/ha), Reklemel (1.13 L/ha) + Vydate C-LV (5.0 L/ha), BIO^ST^ Nematicide 100 (0.026 mg ai/seed), BIO^ST^ Nematicide 100, Reklemel (0.28 L/ha) + Vydate C-LV (1.24 L/ha), BIO^ST^ Nematicide 100 (0.026 mg ai/seed), Reklemel (0.56 L/ha) + Vydate C-LV (2.5 L/ha), BIO^ST^ Nematicide 100 (0.026 mg ai/seed), Reklemel (1.13 L/ha) + Vydate C-LV (5.0 L/ha).

The two-way factorial ANOVA analysis indicated gall ratings were significant for cotton variety response only. Gall ratings conducted 40 DAP showed significantly less galling (*P* < 0.001) on PHY 360 (R) with a 3.4 gall rating compared to 4.7 rating on PHY 340 (S) (data not shown). Final gall ratings at plant harvest increased over the season; however, the severity of the galling remained lower (*P* < 0.001) on PHY 360 (R) at 6.1 compared to the 7.1 galling for PHY 340 (S). No differences in galling were observed when nematicides were applied. A significant interaction between variety x nematicide was observed for *M. incognita* total eggs and eggs per gram of root. The *M. incognita* population density was highest (*P* < 0.05) on PHY 340 (S), although statistically similar (*P* > 0.05) to the population on PHY 360 (R) when neither cultivar was treated with a nematicide (Fig. 2). PHY 340 (S) and PHY 360 (R) without a nematicide, and PHY 340 + Reklemel + Vydate C-LV (0.56 + 2.5 L/ha) supported similar *M. incognita* total population density and eggs per gram of root. The addition of Reklemel + Vydate C-LV at all rates with and without BIO^ST^ Nematicide 100 seed treatment on PHY 360 (R) reduced *M. incognita* population density as compared to PHY 340 (S) without a nematicide but was not enough of a population reduction to separate them from PHY 360 (R) without a nematicide. Reklemel (0.28 and 1.13 L/ha) + Vydate C-LV (1.24 and 5.0 L/ha) without Bio^ST^ Nematicide 100 and all rates of Reklemel + Vydate C-LV with BIO^ST^ Nematicide 100 also reduced *M. incognita* total eggs and eggs per gram of root compared to PHY 340 (S) without a nematicide.

*M. incognita Field 2021*: Similar to the results in 2020, the factorial analysis indicated no significant interactions between cotton cultivars and nematicides for plant parameters. PHY 340 (S) supported similar PS, PH, and RFW compared to PHY 360 (R) (Table 5). The PHY 340 (R) plants had a larger biomass than PHY 360 (R), weighing 3.13 g more on average at 40 DAP. Significant positive correlations between biomass and lint yield (r= 0.14483; *P* < 0.0677) were observed. Lint yield was increased by 524 kg/ha (*P* > 0.05) or 37% when PHY 360 was compared to PHY 340 in 2021.

**Table 5:** Field trial LS means for PHY 360 W3FE [*M. incognita* resistant (R)] and PHY 340 W3FE [*M. incognita* susceptible (S)] cotton variety and nematicide combination effects on plant stand, plant height, root fresh weight, plant biomass, and lint cotton yield at the Plant Breeding Unit, Shorter, AL in 2021.

Source of variation (F-value)	Plant stand (13 m row)	Plant height (cm)	Root fresh weight (g)	Biomass (g)	Lint yield (cotton kg/ha)
Cultivar^a^	0.0334^*b^	0.6081	0.7150	0.0430^**^	0.230^****^
Nematicide^c^	0.2073	0.0001^****^	0.0001^****^	0.0001^***^	0.2907^***^
Cultivar x Nematicide	0.9026	0.7749	0.5254	0.7427	0.6664
**Cultivar LS-means**					
PHY 360 W3FE (R)	81.33^d^ a	16.88 a	3.26 a	42.44 a	1411 a
PHY 340 W3FE (S)	83.69 a	16.73 a	3.22 a	39.31 b	887 b
**Nematicide LS-means**					
No nematicide	82.25 a	15.09 c	2.34 d	30.82 d	964 B
Reklemel (0.21 L/ha) + Vydate C-LV (0.88 L/ha)	82.35 a	16.53 bc	2.96 bcd	37.03 cd	1221 Ab
Reklemel (0.28 L/ha) + Vydate C-LV (1.24 L/ha)	84.00 a	16.41 bc	3.18 bcd	40.07 bcd	1002 Ab
Reklemel (0.56 L/ha) + Vydate C-LV (2.5 L/ha)	81.50 a	17.36 ab	3.41abc	43.25 abc	1171 Ab
BIO^ST^ Nematicide 100^e^ (0.026 mg ai/seed)	84.85 a	14.94 c	2.75 cd	33.15 d	1060 Ab
BIO^ST^ Nematicide 100 (0.026 mg ai/seed) + Reklemel (0.21 L/ha) + Vydate CLV (0.88 L/ha)	82.30 a	17.84 ab	3.71 ab	47.79 ab	1143 Ab
BIO^ST^ Nematicide 100(0.026 mg ai/seed) + Reklemel (0.28 L/ha) + Vydate C-LV (1.24 L/ha)	83.90 a	17.55 ab	3.41 abc	43.78 abc	1316 A
BIO^ST^ Nematicide 100 (0.026 mg ai/seed) + Reklemel (0.56 L/ha) + Vydate C-LV (2.5 L/ha)	78.90 a	18.70 a	4.09 a	51.11 a	1315 A

^a^ All seeds were treated with Metalaxyl 4.0 ST, Fludioxonil 4L ST, Myclobutanil 240 ST, Resonate 600. ^b^ *P*-values for Type III fixed effects with significance at the 0.1, 0.05, 0.01, and 0.001 level is indicated by *, **, ***, and **** respectively. ^c^ Reklemel and Vydate C-LV were applied at planting as an in-furrow spray and BIO^ST^ Nematicide 100 was a seed treatment previously added to the seeds. ^d^ Values present are LS-means separated using the Tukey-Kramer method at *P* ≤ 0.05. Values in the same column followed by the same letter do not differ significantly.

Plant stand was similar between the nematicide combinations. The nematicide combinations did significantly affect PH, RFW, biomass, and lint yields. The nematicide combination BIO^ST^ Nematicide 100 (0.026 mg ai/seed) + Reklemel + Vydate C-LV (0.56 + 2.5 L/ha) had significantly taller plants and heavier root fresh weights over the no-nematicide control and the two lowest rates of Reklemel (0.21 and 0.28 L/ ha) + Vydate C-LV (0.88 and 1.24 L/ha) without BIO^ST^ Nematicide 100 and the BIO^ST^ Nematicide 100 seed treatment nematicide alone. Plant biomass was also greater in the BIO^ST^ Nematicide 100, plus all three rates of Reklemel (0.21, 0.28, and 0.56 L/ha) + Vydate C-LV (0.88, 1.24 and 2.5 L/ha) combinations, compared to BIO^ST^ Nematicide 100 alone and the no-nematicide control. The nematicide combinations of BIO^ST^ Nematicide 100 + Reklemel (0.28 and 0.56 L/ha) + Vydate C-LV (1.24 and 2.5 L/ha) supported the largest lint yields, averaging 352 kg/ha or 27% more lint yield compared to the no-nematicide control.

The two-way factorial ANOVA analysis indicated nematode gall ratings at 40 DAP were significant for cotton variety but not nematicide. Gall ratings at 40 DAP indicated significantly less galling (*P* < 0.001) on PHY 360 (R), with a 5.3 gall rating compared to a 5.9 rating for the PHY 340 (S). Gall ratings at harvest were significant for cotton variety response and nematicide combinations. Final gall ratings at plant harvest increased from the 40 DAP ratings, although the severity of the galling remained lower (*P* < 0.001) on PHY 360 (R) at 5.5 compared to the 6.8 galling for PHY 340 (S). The combination of BIO^ST^ Nematicide 100 (0.026 mg ai/seed) + Reklemel + Vydate C-LV (0.56 + 2.5 L/ha) sustained significantly lower final gall rating (*P* < 0.05) at 5.7.

**Figure 3:**
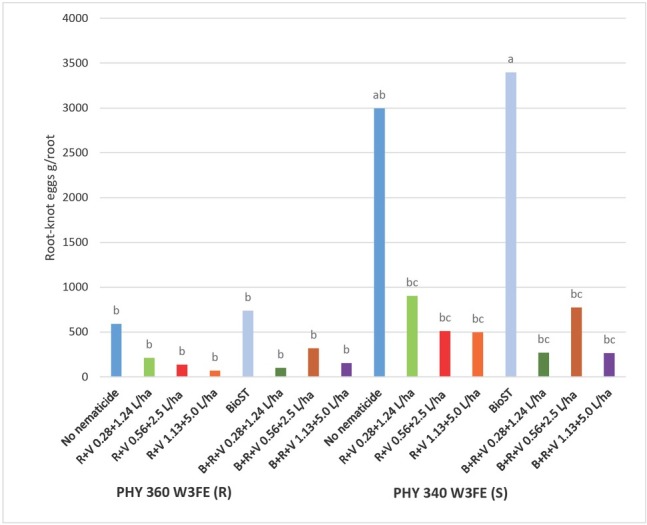
Field trial *M. incognita* eggs per gram of root collected from four root systems LS means for PHY 360 W3FE (R) and PHY 340 W3FE (S) cotton and nematicide combination at 40 DAP in 2021. *P-*value for Type III fixed effects for the Variety x Nematicide interaction was 0.0272. Nematicide treatments included no-nematicide control, Reklemel (0.21 L/ha) + Vydate C-LV (0.88 L/ha) Reklemel (0.28 L/ha) + Vydate C-LV (1.24 L/ha, Reklemel (0.56 L/ha) + Vydate C-LV (2.5 L/ha), BIO^ST^ Nematicide 100 (0.026 mg ai/seed), BIO^ST^ Nematicide 100, Reklemel (0.21 L/ ha) + Vydate C-LV (0.88 L/ha), BIO^ST^ Nematicide 100 (0.026 mg ai/seed), Reklemel (0.28 L/ha) + Vydate C-LV (1.24 L/ha), BIO^ST^ Nematicide 100 (0.026 mg ai/seed), Reklemel (0.56 L/ha) + Vydate C-LV (2.50 L/ha).

A significant interaction was observed between cotton cultivar x nematicides for the nematode parameters *M. incognita* total eggs and *M. incognita* eggs per gram of root (Fig. 3). The PHY 360 (R) supported 61 % and 77 % fewer *M. incognita* total eggs and eggs per gram of root (*P* < 0.05) than PHY 340 (S) with no nematicide treatment. *M. incognita* eggs per gram of root were significantly similar for PHY 360 (R) with or without any of the nematicide combinations.

**Figure 4:**
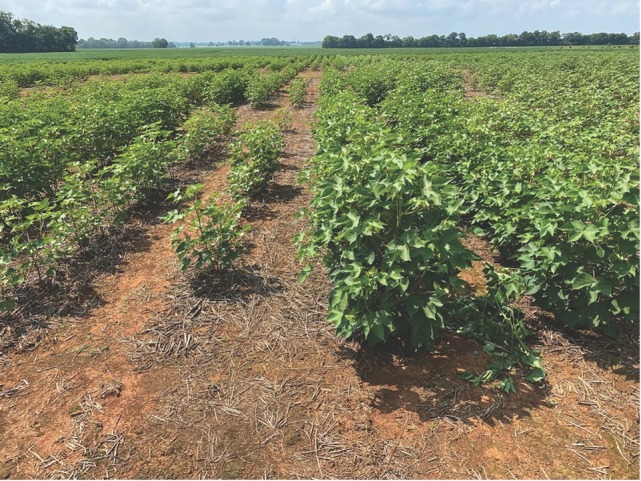
Auburn University’s Tennessee Valley Research Extension Center showing the nematode susceptible PHY 340 W3FE on the left and the *R. reniformis-*resistant PHY 332 W3FE on the right 102 DAP.

*R. reniformis Field 2020*: Data analysis indicated no significant interactions between the cotton cultivars and nematicide for the plant parameters. Plant stand was greater for the resistant PHY 332 (R) with 6.7 plants per meter of row at 30 DAP compared to 5.4 plants per meter of row for the susceptible PHY 340 (S) (Table 6). Plant stand, PH, RFW, biomass, and lint yield were significantly higher with PHY 332 (R) compared to PHY 340 (S). The biomass was increased by 5.2 g at 40 DAP (Fig. 4). Lint yield was 714 kg/ha or 36% greater with PHY 332 (R) compared to PHY 340 (S).

**Table 6:** **Field trial LS means for PHY 332 W3FE [*R. reniformis*** *-* **resistant (R)] and PHY 340 W3FE [*R. reniformis*** *-* **susceptible (S)] cotton variety and nematicide combination effects on plant stand, plant height, root fresh weight, plant biomass, and lint cotton yield at the Tennessee Valley Research and Extension Center, Belle Mina, AL in 2020.**

Source of variation (F-value)	Plant stand (13 m row)	Plant height (cm)	Root fresh weight (g)	Biomass (g)	Lint cotton yield (kg/ha)
Cultivar^a^	0.0001^****b^	0.0139^***^	0.0004^****^	0.0001^****^	0.0001^****^
Nematicide^c^	0.0732^*^	0.0001^***^	0.0001^****^	0.0001^***^	0.0001^****^
Cultivar x Nematicide	0.0966	0.3482	0.9314	0.9473	0.1298
**Cultivar LS-means**					
PHY 332 W3FE (R)	51.20^d^ a	13.42 a	2.52 a	21.82 A	1998 b
PHY 340 W3FE (S)	41.34 b	12.42 b	1.97 b	16.62 B	1284 a
**Nematicide LS-means**					
No nematicide	51.20 a	11.73 c	1.72 c	14.32 B	1289 c
Reklemel (0.28 L/ha) + Vydate C-LV (1.24 L/ha)	46.85 a	13.33 abc	2.14 abc	19.29 ab	1545 ab
Reklemel (0.56 L/ha) + Vydate C-LV (2.5 L/ha)	50.35 a	14.10 a	2.64 a	23.70 A	1732 ab
Reklemel (1.13 L/ha) + Vydate C-LV (5.0 L/ha)	46.70 a	12.63 abc	1.98 bc	18.13 ab	1668 ab
BIO^ST^ Nematicide 100^e^ (0.026 mg ai/seed)	46.80 a	12.36 bc	2.1 abc	18.08 ab	1504 bc
BIO^ST^ Nematicide 100 (0.026 mg ai/seed) + Reklemel (0.28 L/ha) + Vydate C-LV (1.24 L/ha)	40.20 a	12.59 abc	2.12 abc	17.67 ab	1687 ab
BIO^ST^ Nematicide 100(0.026 mg ai/seed) + Reklemel (0.56 L/ha) + Vydate C-LV (2.5 L/ha)	45.10 a	13.45 ab	2.71 a	21.95 A	1777 a
BIO^ST^ Nematicide 100 (0.026 mg ai/seed) + Reklemel (1.13 L/ha) + Vydate C-LV (5.0 L/ha)	42.95 a	13.16 abc	2.57 ab	20.60 A	1731 ab

^a^All seeds were treated with Metalaxyl 4.0 ST, Fludioxonil 4L ST, Myclobutanil 240 ST, Resonate 600. ^b^*P*-values for Type III fixed effects with significance at the 0.1, 0.05, 0.01, and 0.001 level are indicated by *, **, ***, and ****, respectively. ^c^Reklemel and Vydate C-LV were applied at planting as an in-furrow spray and BIO^ST^ Nematicide 100 was a seed treatment previously added to the seeds. ^d^Values present are LS-means separated using the Tukey-Kramer method at *P* ≤ 0.05. Values in the same column followed by the same letter do not differ significantly.

The addition of the nematicides did not have a significant effect on plant stand in the *R. reniformis* field. The nematicide combination of Reklemel + Vydate C-LV (0.56 + 2.5 L/ha) with and without BIO^ST^ Nematicide 100 produced the tallest cotton plants (*P* < 0.05) as compared to the no-nematicide control. Root fresh weight and biomass were significantly increased with Reklemel + Vydate C-LV (0.56 + 2.5 L/ha) with or without BIO^ST^ Nematicide 100 as compared to the no-nematicide control. All nematicide combinations of Reklemel + Vydate C-LV at all rates significantly supported more cotton lint yield than the no-nematicide control. Lint yield was increased by 488 kg/ha or 27% with the addition of the nematicide combination BIO^ST^ Nematicide 100 + Reklemel + Vydate C-LV (0.56 + 2.5 L/ha) compared to the no-nematicide group.

**Figure 5:**
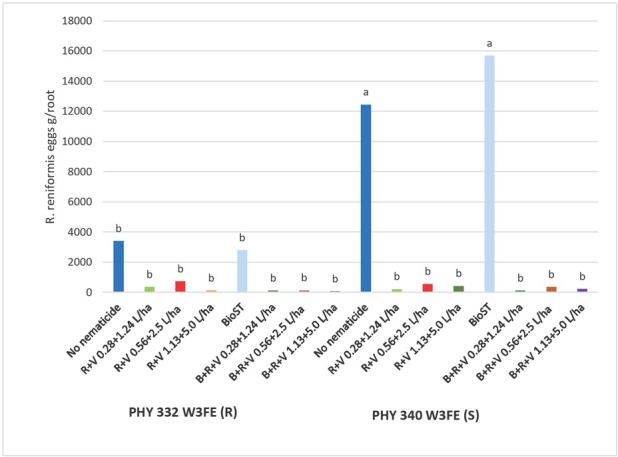
Field trial *R. reniformis* eggs per gram of root collected from four root systems LS means for PHY 332 W3FE (R) and PHY 340 W3FE (S) cotton and nematicide combination at 40 DAP in 2020. *P-*value for Type III fixed effects for the Variety x Nematicide interaction was 0.0178. Nematicide treatments included no-nematicide control, Reklemel (0.28 L/ha) + Vydate C-LV (1.24 L/ha), C-LV (0.28 +1.24 L/ha), Reklemel (0.56 L/ha) + Vydate C-LV (2.5 L/ha), Reklemel (1.13 L/ha) + Vydate C-LV (5.0 L/ha), BIO^ST^ Nematicide 100 (0.026 mg ai/seed), BIO^ST^ Nematicide 100, Reklemel (0.28 L/ha) + Vydate C-LV (1.24 L/ha), BIO^ST^ Nematicide 100 (0.026 mg ai/seed), Reklemel (0.56 L/ha) + Vydate C-LV (2.5 L/ha), BIO^ST^ Nematicide 100 (0.026 mg ai/seed), Reklemel (1.13 L/ha) + Vydate C-LV (5.0 L/ha).

A significant interaction was observed between cotton cultivar x nematicides for the nematode parameters for *R. reniformis* total numbers as well as the ratio of eggs per gram of root (Fig. 5). The PHY 340 (S) alone and PHY 340 (S) + BIO^ST^ Nematicide 100 supported high *R. reniformis* total numbers and eggs per gram of root (12,438 and 15,683 eggs per g/ root, respectively). The *R. reniformis* total egg number density and eggs per gram of root numbers for PHY 332 (R) were 57% and 73% lower, respectively (*P* < 0.05) than on PHY 340 (S). Addition of the nematicides Reklemel + Vydate C-LV at all rates significantly (*P* < 0.05) reduced *R. reniformis* total egg counts and eggs per gram of root with PHY 340 (S). The addition of nematicides did not significantly further reduce the *R. reniformis* populations of PHY 332 (R).

*R. reniformis Field 2021*: In 2021, the two-way factorial ANOVA analysis indicated no significant interactions between the cotton cultivar and nematicide for the plant parameters, similar to the observations in 2020 (Table 7). Plant stand was greater for PHY 332 (R) with 5.3 plants per meter of row compared to 3.2 plants per meter of row for PHY 340 (S). Cotton cultivar did not impact PH or RFW. Total plant biomass was greater (*P* < 0.05) with PHY 332 (R) compared to PHY 340 (S). Lint yield was increased (*P* < 0.001) by 703 kg/ha or 42% when growing the resistant PHY 332 (R) compared to PHY 340 (S) in 2021.

**Table 7:** Field trial LS means for PHY 332 W3FE [*R. reniformis-*resistant (R)] and PHY 340 W3FE [*R. reniformis-*susceptible (S)] cotton variety and nematicide combination effects on plant stand, plant height, root fresh weight, plant biomass, and lint cotton yield at the Tennessee Valley Research and Extension Center, Belle Mina, AL in 2021.

Source of variation (F-value)	Plant stand (13 m row)	Plant height (cm)	Root fresh weight (g)	Biomass (g)	Lint cotton yield (kg/ha)
Cultivar^a^	0.0001^****b^	0.8351	0.1794	0.0207^**^	0.0001^****^
Nematicide^c^	0.0209^**^	0.0001^***^	0.0001^****^	0.0001^****^	0.0001^****^
Cultivar x Nematicide	0.1123	0.9458	0.7584	0.7887	0.8616
**Cultivar LS-means**					
PHY 332 W3FE (R)	40.85^d^ a	11.22 a	1.20 a	12.46 A	1692 a
PHY 340 W3FE (S)	24.81 b	12.42 a	1.10 a	11.07 B	989 b
**Nematicide LS-means**					
No nematicide	30.07 ab	9.68 c	0.74 c	8.07 C	1008 c
Reklemel (0.21 L/ha) + Vydate C-LV (0.88 L/ha)	39.62 a	10.61 abc	1.10 abc	abc 10.90	1253 bc
Reklemel (0.28 L/ha) + Vydate C-LV (1.24 L/ha)	34.26 ab	11.64 ab	1.30 a	12.96 ab	1456 ab
Reklemel (0.56 L/ha) + Vydate C-LV (2.5 L/ha)	35.60 ab	11.48 abc	1.32 a	12.49 ab	1445 ab
BIO^ST^ Nematicide 100^e^ (0.026 mg ai/seed)	32.43 ab	10.28 bc	0.87 bc	9.30 bc	1099 c
BIO^ST^ Nematicide 100 (0.026 mg ai/seed) + Reklemel (0.21 L/ha) + Vydate C-LV (0.88 L/ha)	32.37 ab	12.15 a	1.33 a	13.55 A	1334 abc
BIO^ST^ Nematicide 100(0.026 mg ai/seed) + Reklemel (0.28 L/ha) + Vydate C-LV (1.24 L/ha)	30.19 ab	12.00 ab	1.35 a	14.23 A	1538 ab
BIO^ST^ Nematicide 100 (0.026 mg ai/seed) + Reklemel (0.56 L/ha) + Vydate C-LV (2.5 L/ha)	28.10 b	11.63 ab	1.20 ab	12.62 ab	1591 a

^a^All seeds were treated with Metalaxyl 4.0 ST, Fludioxonil 4L ST, Myclobutanil 240 ST, Resonate 600. ^b^*P*-values for Type III fixed effects with significance at the 0.1, 0.05, 0.01, and 0.001 level are indicated by *, **, ***, and ****, respectively. ^c^Reklemel and Vydate C-LV were applied at planting as an in-furrow spray and BIO^ST^ Nematicide 100 was a seed treatment previously added to the seeds. ^d^Values present are LS-means separated using the Tukey-Kramer method at *P* ≤ 0.05. Values in the same column followed by the same letter do not differ significantly.

The lowest rate nematicide combination of Reklemel + Vydate C-LV (0.21 L/ha + 0.88 L/ha) supported a plant stand of 5.2 plants per meter of row as compared to BIO^ST^ Nematicide 100 + Reklemel + Vydate C-LV (0.56 + 2.5 L/ha), which supported 3.7 plants per meter of row. Plant height was significantly increased with the nematicide combination BIO^ST^ Nematicide 100 + Reklemel (0.21 L/ha) + Vydate C-LV (0.88 L/ha) as compared to the BIO^ST^ Nematicide 100 alone and the no-nematicide control. Root fresh weight was increased in the nematicide combinations of Reklemel (0.21 or 0.28 L/ha) + Vydate C-LV (0.88 or 1.24 L/ha) with or without BIO^ST^ Nematicide 100 seed treatment as compared to the no-nematicide control. Similarly, plant biomass was also significantly larger in the Reklemel (0.21 or 0.28 L/ha) + Vydate C-LV (0.88 or 1.24 L/ha) but only when combined with BIO^ST^ Nematicide 100 seed treatment as compared to the no-nematicide control. Lint cotton yields were greater in the nematicide combination of Reklemel + Vydate C-LV (0.56 + 2.5 L/ha) in combination with BIO^ST^ Nematicide 100 than the BIO^ST^ Nematicide 100 alone and the no-nematicide control. Lint yield was increased (*P* < 0.001) by 583 kg/ha or 37% when using nematicide combination BIO^ST^ Nematicide 100 Reklemel + Vydate C-LV (0.56 + 2.5 L/ha) compared to no-nematicide. The nematicide combinations of Reklemel (0.28 and 0.56L/ha) + Vydate C-LV (1.24 + 2.5 L/ha) with or without BIO^ST^ Nematicide 100 seed treatment produced lint cotton yields that were (*P* < 0.05) similar, varying by 146 kg/ha.

**Figure 6:**
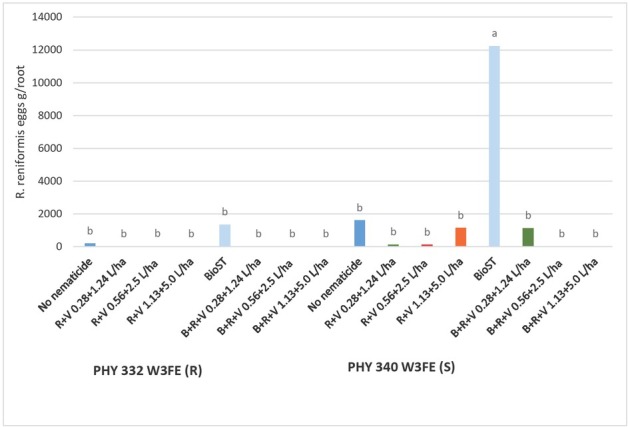
Field trial *R. reniformis* eggs per gram of root collected from four root systems. LS means for PHY 332 W3FE (R) and PHY 340 W3FE (S) cotton and nematicide combination at 40 DAP in 2021. *P-*value for Type III fixed effects for the Variety x Nematicide interaction was 0.0441. Nematicide treatments included no-nematicide control, Reklemel (0.28 L/ha) + Vydate C-LV (1.24 L/ha), C-LV (0.28 +1.24 L/ha), Reklemel (0.56 L/ha) + Vydate C-LV (2.5 L/ha), Reklemel (1.13 L/ha) + Vydate C-LV (5.0 L/ha), BIO^ST^ Nematicide 100 (0.026 mg ai/seed), BIO^ST^ Nematicide 100, Reklemel (0.28 L/ha) + Vydate C-LV (1.24 L/ha), BIO^ST^ Nematicide 100 (0.026 mg ai/seed), Reklemel (0.56 L/ha) + Vydate C-LV (2.5 L/ha), BIO^ST^ Nematicide 100 (0.026 mg ai/seed), Reklemel (1.13 L/ha) + Vydate C-LV (5.0 L/ha).

A significant interaction was observed between the cultivar and nematicides in the presence of *R. reniformis* for total population density and eggs per gram of root (Fig. 6). PHY 332 (R) nematode density and eggs per gram of root with and without nematicide applications were significantly lower than the *R. reniformis* population on PHY 340 (S) with no nematicide or with BIO^ST^ Nematicide 100 nematicide. The PHY 340 (S) + BIO^ST^ Nematicide 100 supported the highest *R. reniformis* total population density and eggs per gram of root. The addition of all rates of Reklemel + Vydate C-LV with or without BIO^ST^ Nematicide 100 nematicide combinations significantly reduced *R. reniformis* total eggs and eggs per gram of root with both the PHY 340 (S) and PHY 332 (R) as compared to the PHY 340 (S) with or without BIO^ST^ Nematicide 100.

## Discussion

The primary goal of this study was to determine if the cultivars PHY 360 (R) and PHY 332 (R) would support lower nematode population density and produce a higher cotton yield compared to the nematode-susceptible cultivar PHY 340 (S) in fields with a history of high nematode pressure. In the greenhouse setting with *M. incognita* and *R. reniformis*, the resistant cultivars were effective at suppressing nematode population density when compared to a susceptible cultivar. Each year of the experiments, new seeds of each cultivar were tested and, in both years, the PHY 360 (R) and PHY 332 (R) supported less than 25% of the populations that developed on the susceptible PHY 340 cultivar.

Results of our field experiments confirm previous reports that PHY 340 (S) is susceptible and serves as a good host to *M. incognita* and *R. reniformis* nematodes, while indicating that the new resistant cultivars PHY 360 (R) and PHY 332 (R) are effective at keeping nematode populations low. Each year of the experiments, new seeds of each cultivar were assessed and, in both years, the PHY 360 (R) and PHY 332 (R) supported less than 35% and first 20%, respectively, of the populations that developed on the susceptible PHY 340 cultivar. Considerable progress has been made since the initial introduction of the first commercial cotton cultivar LA887, which was registered with partial resistance to *M. incognita* (Jones et al., 1991; Wheeler et al., 2020). Breeding efforts have been working toward the commercial release of resistant cultivars including the initial breeding with the *G. hirsutum* species by Starr et al., (2007) and Weaver et al., (2007) and with *G. longicalyx* by Robinson et al. (2007) and Bell et al. (2014). In our experiments, PHY 332 suppressed *R. reniformis* populations while yielding more cotton than the susceptible PHY 340, which is an improvement over the initial hypersensitive experience by the first *R. reniformis* genotypes (Schrimsher et al. 2014). These resistant cultivars supported fewer nematodes while producing optimum cotton yields. Additionally, as seen in previous research, nematode-resistant cotton genotypes have been shown to reduce nematode reproductive potential for *M. incognita* and *R. reniformis* (Koenning et al., 1996; Schrimsher et al., 2014). We observed the reduction in nematode populations on these newly released cotton cultivars. These new cotton cultivars have improved from the first PHY 417 WR cotton cultivar, which had high resistance to *M. incognita* but poor yield potential and thus had limited commercial adaptation (Fuchs et al., 2015; Wheeler et al., 2020).

The secondary goal was to evaluate the potential of the addition of BIO^ST^ Nematicide 100 seed treatment nematicide with the addition of Reklemel and Vydate C-LV in-furrow spray nematicides to the resistant cotton cultivars PHY 360 and PHY 332 to further manage *M. incognita* and *R. reniformis* nematodes in cotton. Of these nematicides, BIO^ST^ Nematicide 100 and Vydate C-LV (Oxamyl) are registered for use on cotton; however, Reklemel (Corteva Agriscience, Indianapolis, IN) is currently registered for use on fruits and vegetables (Desaeger et al., 2020). Results of our field experiments indicate applying BIO^ST^ Nematicide 100 as a seed treatment or BIO^ST^ Nematicide 100 + Reklemel + Vydate C-LV in-furrow nematicides to PHY 360 and PHY 332 further lowered nematode population density. This research agrees with previous findings with different nematicides that applying nematicides to manage *M. incognita* and *R. reniformis* nematodes reduces populations and often enhances yields of nematode-resistant cotton genotypes and now cultivars (Lawrence and McLean, 2000; Wheeler et al., 2013; Schrimsher et al., 2014). The addition of nematicides with the genetic resistance of the cotton cultivars will be a two-method integrated nematode management program to prevent the development of nematodes that can overcome the resistance genes in the new cotton cultivars, thus allowing for a longer production life of these cultivars.

*M. incognita*: At PBU overall, the resistant PHY 360 variety produced 21% more lint cotton than the susceptible variety. The combination of the low and mid-rate of BIO^ST^ Nematicide 100 + Reklemel (0.28 and 0.56 L/ha) + Vydate C-LV (1.24 and 2.5 L/ ha) supported the lowest total eggs and eggs per gram of root. These findings are consistent with Desaeger et al. (2020), where applications of Reklemel and Vydate C-LV significantly reduced *M. hapla* on strawberry and *M. javanica* in tomato. Reklemel has also been shown to reduce *M. incognita* populations on carrot (Becker, 2022) and cucumber. The use of the nematicide seed treatment BIO^ST^ Nematicide 100 overall produced a growth benefit to the cotton plants with increased plant height, root fresh weight, and plant biomass. PHY 340 (S) supported the largest root fresh weight, potentially due to the galling caused by the large *M. incognita* population density. Chitwood et al. (1952) reported an increase in root fresh weight with larger populations of *M. incognita* in peach rootstocks.

The nematicide combination that supported the largest lint yield was the BIO^ST^ Nematicide 100 + Reklemel (0.56 L/ha) + Vydate C-LV (2.5 L/ha). A similar study done by Koenning et al. (2004) indicated that increasing nematicide rate led to increased lint yields. We observed that the combination of nematicides applied as a seed treatment with an additional in-furrow treatment at planting increased lint yield an average of 26% when compared to no nematicide, while adding Reklemel + Vydate alone increased lint yield 17% compared to no nematicide. An application of BIO^ST^ Nematicide 100 increased yield by 8% alone. A trial conducted by Dyer et al. (2016) supports this conclusion and found that nematicide seed treatment BIO^ST^ Nematicide 100 increased lint yield ranging from 1% to 12%, depending on the rate of the seed treatment.

*R. reniformis*: At TVREC overall, the PHY 332 (*R. reniformis* R) cultivar produced 39% more lint cotton than the susceptible variety. The use of the nematicide seed treatment BIO^ST^ Nematicide 100 overall produced a growth benefit to the cotton plants with increased plant height, root fresh weight, and plant biomass similar to the plant growth observed in the *M. incognita* trial. Biological products have been shown to increase plant growth while reducing nematode numbers. Xiang et al. (2018) established the utilization of specific *Bacillus* spp. reduced *M. incognita* population density in cotton. The cultivar PHY 332 (R) and the nematicide combination of BIO^ST^ Nematicide 100 + Reklemel (0.28 L/ha) + Vydate C-LV (1.24 L/ha) was the most effective in lowering *R. reniformis* total eggs and eggs per gram of root. This combination also produced the highest lint yield. Research supports the use of Vydate C-LV (Lawrence et al., 2007) on reducing *R. reniformis* population levels and Reklemel reducing *M. incognita* populations (Lahm et al., 2017). An application of the seed treatment nematicide BIO^ST^ Nematicide 100 increased yield by 12% overall tests compared to the no-nematicide treatment. The in-furrow nematicides, Reklemel + Vydate C-LV average over all tests and rates increased lint cotton yield an average of 21%. However, the combination; BIO^ST^ Nematicide 100 + Reklemel + Vydate C-LV lint yield increased 28% as compared to the no-nematicide control. Previous experiments conducted by Dyer et al. (2016) combined BIO^ST^ Nematicide 100 with other plant nematicides and found an increase in subsequent yields. A study done by Zimet et al. (2002) confirms that there are economic benefits to applying nematicides such as 1,3-D and aldicarb to increase cotton lint yields in the presence of plant-parasitic nematodes. Gordon et al. (2022) most recently found an economic yield increase when adding fluopyram seed treatment nematicides to high-yielding susceptible cotton cultivars in *M. incognita*- and *R. reniformis*-infested cotton fields. This research enhances previous studies that have shown a yield benefit to tolerant cultivars with an addition of a nematicide in cotton (Dyer et al., 2020). High-yielding nematode-resistant cotton cultivars are the most desirable form of nematode management. Continued integrated nematode management research is needed to determine the durability of resistance in these long-awaited *M. incognita*- and *R. reniformis*-resistant cotton cultivars (Young, 1992).

## Conclusion

Overall, *M. incognita*-resistant cultivar PHY 360 and *R. reniformis*-resistant PHY 332 supported higher yield potential than the nematode-susceptible PHY 340 in nematode-infested fields. It can be concluded from these two-year studies that the genetic resistance in these cultivars repressed nematode population development in fields with a history of high nematode populations. In conclusion, growing these *M. incognita* and *R. reniformis* nematode-resistant cotton cultivars in fields infested with *M. incognita* and *R. reniformis* will produce higher yields than the susceptible cultivars available at this time. In addition, resistant cotton genotypes may suppress *M. incognita* and *R. reniformis* populations for future growing seasons. Combining the nematicides Reklemel, Vydate C-LV, and BIO^ST^ Nematicide 100 with genetic resistance under nematode pressure did increase lint yield in the presence of *M. incognita* and *R. reniformis*. To our knowledge, this is the first published study on Reklemel and Vydate C-LV efficacy as an in-furrow plant cotton nematicide combination. The combination of the resistant cultivars and the application of the biological and chemical nematicides combined into an integrated nematode management system appears to have strong potential for sustaining high yields while reducing nematode populations.
